# Emergence of New Delhi Metallo-Beta-Lactamase (NDM) and* Klebsiella pneumoniae *Carbapenemase (KPC) Production by* Escherichia coli *and* Klebsiella pneumoniae *in Southern Vietnam and Appropriate Methods of Detection: A Cross-Sectional Study

**DOI:** 10.1155/2019/9757625

**Published:** 2019-04-23

**Authors:** Cuong Q. Hoang, Hai D. Nguyen, Huy Q. Vu, Anh T. Nguyen, Binh T. Pham, Trung L. Tran, Hanh T. H. Nguyen, Y. M. Dao, Tuyet S. M. Nguyen, Dung A. Nguyen, Hang T. T. Tran, Lan T. Phan

**Affiliations:** ^1^The Pasteur Institute, Ho Chi Minh City 700000, Vietnam; ^2^Department of Medical Laboratory Science, Faculty of Nursing and Medical Technology, University of Medicine and Pharmacy, Ho Chi Minh City 700000, Vietnam; ^3^Molecular Biomedical Center for Diagnosis and Training, University Medical Center Branch No. 2, Medical and Pharmacy University Hospital, Ho Chi Minh City 700000, Vietnam; ^4^College of Dentistry, Yonsei University, 50-1 Yonsei-ro, Seodaemun-gu, Seoul 03722, Republic of Korea; ^5^Department of Microbiology, Dong Nai General Hospital, Dong Nai Province 710000, Vietnam; ^6^Department of Microbiology, Gia Dinh People's Hospital, Ho Chi Minh City 700000, Vietnam

## Abstract

Carbapenemase-producing Enterobacteriaceae (CPE) are well known to cause many serious infections resulting in increasing mortality rate, treatment cost, and prolonged hospitalization. Among the widely recognized types of carbapenemases, New Delhi *β*-lactamase (NDM) and* Klebsiella pneumoniae *carbapenemase (KPC) are the most important enzymes. However, in Vietnam, there are only scattered reports of CPE due to the lack of simple and affordable methods that are suitable to laboratory conditions. This study aims to survey the characteristics of carbapenem-resistant* E. coli* and* K. pneumoniae* (CR-E/K) at two hospitals in Southern Vietnam and perform some simple methods to detect the two enzymes. A total of 100 CR-E/K strains were collected from clinical isolates of Gia Dinh People's Hospital and Dong Nai General Hospital, Vietnam, from November 2017 to May 2018. The patient-related information was also included in the analysis. We conducted real-time polymerase chain reaction (PCR), Modified Hodge Test (MHT), and combined disk test (CDT) on all isolates. Carbapenemase-encoding genes were detected in 47 isolates (36 NDM, 10 KPC, and one isolate harboring both genes). The* E. coli *strain carrying simultaneously these two genes was the first case reported here. Most of isolates were collected from patients in ICU, Infectious Disease Department, and Department of Urologic Surgery. Urine and sputum were two common specimens. The true positive rate (sensitivity, TPR) and specificity (SPC) of the imipenem–EDTA (ethylen diamine tetra acetic acid) for NDM detection and the imipenem–PBA (phenylboronic acid) for KPC detection on* E. coli *were 93.8%, 97.1% and 66.7%, 95.7%, respectively. Meanwhile, the imipenem–EDTA for NDM detection and the imipenem–PBA for KPC detection among* K. pneumonia* achieved 90.5%, 100% and 100%, 92.9% TPR and SPC, respectively. However, MHT showed low sensitivity and specificity. Our findings showed that CP-E/K were detected with high prevalence in the two hospitals. We suggest that CDT can be used as a low-priced and accurate method of detection.

## 1. Introduction

Antibiotic resistance is a tremendous health problem. This includes the resistance to carbapenems which was considered as the last resort for Enterobacteriaceae infections [1]. In 2017, the World Health Organization published a list of superbugs including carbapenem-resistant Enterobacteriaceae. Carbapenemase production and ESBL/AmpC *β*-lactamase production coupled with porin loss or efflux pump were the common ways that Enterobacteriaceae become resistant to carbapenems [2]. However, the carbapenemase production was more dangerous than the others [2].* E. coli *and* K. pneumoniae *are the most common pathogens in Enterobacteriaceae family. These carbapenemasae-producing bacteria were found in many countries, such as China [3], Parkistan [4], India [5], Turkey [6], Brazil [7], Mexico [8], Peru [9], and Greece [10]. Through this mechanism, New Delhi Metallo-*β*-lactamase-1 (NDM-1) was found in Asia with the highest frequency;* Klebsiella pneumoniae *carbapenemase (KPC) was the most popular enzyme causing carbapenem resistance, especially KPC-2 [1, 11, 12]. Furthermore, KPC-producing bacteria have spread all over the world, including Asia [11]. Some gray literature also reported about KPC-producing Enterobacteriaceae in Vietnam [13–15]. In other important views, these two genes harboring bacteria were more dangerous and resistant to carbapenem with a higher level than bacteria that harbored other types of carbapenemase-encoding genes (including OXA-48-like, another popular carbapenemase present in Enterobacteriaceae) [6, 16–18]. For instance, these carbapenemase-producing bacteria result in severe infections with high mortality rate; the infection caused by KPC-producing* K. pnuemoniae *was from 40% to 56% [16] and up to 88% for NDM-1 producing Enterobacteriaceae [6]. Carbapenem minimum inhibitory concentrations (MICs) for these two types of carbapeenemase producers in recent studies were higher than carbapenem MIC values against OXA-48 type producers [17, 18].

In another aspect, these bacteria have spread rapidly through mobilisable genetic elements [19, 20], for example, plasmid IncX3 [21], IncA/C2 [22]; transposon Tn*4401 *[23], Tn*125* [21], or class I Integron [24]. These elements also play important roles in the existence of multiple genes and transporting various multidrug-resistant genes between bacterial species.

In Vietnam, the prevalence of these CPE is increasingly being reported in the hospital and aquatic environment [25–27]. Data about carbapenemase-producing* E. coli *and* K. pneumoniae* were reported notably in Southern and Northern Vietnam. Due to the lack of needed methods for screening and detection, there is an underestimation of CPE in other regions of Vietnam. The requirement for low-priced and efficient methods to screen or confirm carbapenemase-producing bacteria in laboratories, which are likely to be suitable for low-resource settings in Vietnam, is urgent. Today, the combinations of meropenem and varbobactam or imipenem and relebactam are taken into consideration as an option to treat infections caused by KPC-producing organisms [28, 29]. Therefore, it is vital to select an efficient method to detect and characterize these carbapenemase-producing Enterobacteriaceae, particularly in Vietnam.

Among various methods, PCR, real-time PCR, and DNA sequencing are the gold standards for carbapenemase-encoding genes detection, but these methods have not been widely used in Vietnam due to the high cost. Phenotypic tests such as Modified Hodge Test and the combined disk test are suitable because of the lower cost. The combined disk test is based on the synergy between metallo-*β*-lactamases (MBLs) or KPC inhibitors (EDTA or PBA) and carbapenems. Many studies used these methods but none of them investigated* E. coli *and* K. pneumoniae *separately [30, 31]. The purpose of this study was to evaluate the characteristics of NDM/KPC-producing* E. coli/K. pneumoniae* at the hospitals in the South Vietnam and to assess some simple methods for detecting these bacteria in laboratory conditions.

## 2. Materials and Methods

### 2.1. Study Design and Sample Collection

The study was designed as a cross-sectional study, including all clinical isolates from November 2017 to May 2018. The study was conducted at the Molecular Biomedicine Laboratory in the Department of Medical Laboratory Science, University of Medicine and Pharmacy, Ho Chi Minh City. 100 clinical isolate strains (50* E. coli *and 50* K. pneumoniae*) were collected from the Microbiology Department of Gia Dinh People's Hospital and Dong Nai General Hospital. All of them were nonsusceptible to one of the carbapenems (imipenem, meropenem, or ertapenem) detected by the automatic systems. Patient data such as age, sex, specimen types, and clinical wards were gathered to survey the characteristics of patients. Sample list and relevant information are supplied in Annex 1.

### 2.2. Bacterial Isolates

Fifty-nine isolates from Gia Dinh People's Hospital were identified and subjected to antimicrobial susceptibility testing by using the Vitek 2 system (bioMérieux Vitek Inc., Hazelwood, MO, USA). Furthermore, ID/AST of 41 isolates from Dong Nai General Hospital was performed by using BD Phoenix™ (USA). From those hospitals, a single colony obtained from pure culture was inoculated into BHI (Heart Infusion Broth, Himedia, India) broth, with 20% glycerol (Xilong medical, China), and stored at -20°C, to reculture for extracting DNA for real-time PCR and performing phenotypic methods.

### 2.3. Real-Time PCR

#### 2.3.1. DNA Extraction

Bacterial DNA was extracted by heat treatment. One colony form pure isolates incubated overnight on MacConkey agar (Merck KgaA, Darmstadt, Germany) was suspended in 200*μ*L of Tris-EDTA pH 8.0. The suspension was heated at 95°C for 20 minutes, followed by centrifugation at 13,000 revolutions per minute for 10 minutes to collect 150*μ*L supernatant, and stored at -20°C for real-time PCR. DNA quality was evaluated using NanoDrop™ 2000 Spectrophotometer (ThemoFisher Scientific, Wilmington, Delaware, USA) with the acceptable range for the ratio of absorbance at 260 nm/280 nm from 1.6 to 1.8.

#### 2.3.2. Genotypic Detection of blaNDM, blaKPC, and 16S rRNA

The genes were detected by real-time PCR using TaqMan probes for all samples and were confirmed by DNA sequencing of selected positive samples. The bacterial DNA was performed to screen for the presence of* bla*NDM and* bla*KPC. The reaction used bacterial 16S rRNA gene as an internal control for DNA extraction and amplification process. The primers and probes used for* bla*NDM,* bla*KPC, and 16S rRNA were described by the Center of Disease Control and Prevention [32]. To carry out real-time PCR, 5*μ*L DNA was added to a final volume of 20*μ*L Master Mix, which included 1X PCR buffer, 4 mM MgCl_2_, 0.2 mM dNTPs, and 5 IU Taq DNA polymerase (Solgent Co., Ltd., South Korea). The concentration for each primers and probes is illustrated in Table 1. The negative control for PCR reaction is sterile DEPC-treated water. DNA from GD018, which is a clinical isolate that carries both* bla*NDM and* bla*KPC (confirmed by DNA sequencing), is used as a positive control.

The real-time PCR conditions were chosen as follows: Enzyme activation was achieved by primary heating step at 95°C for 15 minutes. This was followed by 40 cycles of 95°C for 15 seconds and 56°C for 20 seconds. Real-time PCR was run on CFX96 Touch™ Real-Time PCR Detection System (Bio-Rad, Hercules, California, USA). Fluorescence signal was detected after each cycle. Positive sample was determined when the automatically normalized fluorescence signal rises above the threshold limit value calculated in each run. A total of 40 cycles takes 70 minutes.

#### 2.3.3. DNA Sequencing

DNA extracts from selected positive samples for* bla*NDM (GD013, GD002, and GD018) and* bla*KPC (GD011, GD16, and GD018) were sequenced using the sequencing primers. Initial PCR proceeded in the above-mentioned conditions, and PCR products were sent to 1-BASE (Singapore) for Sanger's sequencing. 16S rRNA genes from two samples GD013 and GD018 were also sequenced to confirm the validity of the internal control primers.

### 2.4. Modified Hodge Test (MHT)

The MHT was carried out on all isolates, following the Clinical & Laboratory Standards Institute (CLSI) guide on Mueller-Hinton agar (MHA–Becton Diskinson, US) using 10*μ*g meropenem (MEM) disks (Nam Khoa Biotech, Vietnam) [33].* Klebsiella pneumoniae *ATCC®BAA–1705 and* Klebsiella pneumoniae *ATCC®BAA–1706 were used as a positive control and a negative control, respectively.

### 2.5. Combined Disk Test (PBA/IPM–EDTA/IPM)

Phenylboronic acid solution was prepared by dissolving 2g phenylboronic acid (PBA) (Sigma-Aldrich, Steinheim, Germany) in 100mL solution containing 50mL distilled water and 50mL Dimethyl Sulfoxide (Xilong medical, China). EDTA 0.25M solution prepared by mixing 18.61g disodium ETDA.2H_2_O in 200 mL of distilled water was adjusted to pH 8.0 by using NaOH. Both solutions were autoclaved at 121°C for 15 minutes. Either 20*μ*L phenylboronic acid solution (400*μ*g PBA) or 10*μ*L EDTA solution (930*μ*g EDTA) was added on a 10*μ*g imipenem disk (IPM) to get a PBA/IPM or EDTA/IPM disk, respectively. These disks were used within 1 hour.

A bacterial suspension with McFarland Equivalence Turbidity Standard 0.5 was streaked on MHA plate using a swab. Three disks, IPM, EDTA/IPM, and PBA/IPM, were placed on the MHA plate with the distance from one disk to another being at least 24 mm.* Klebsiella pneumoniae *ATCC®BAA–1705 and* Klebsiella pneumoniae *ATCC®BAA–1706 were used as a positive control and a negative control, respectively. An increase of 7 mm for EDTA/IPM or 5 mm for PBA/IPM in the diameter of the zone of inhibition compared to that of IPM was considered as a positive result for NDM or KPC production, respectively.

### 2.6. Statistical Analysis

Data were entered using Excel 2010, and all statistical analyses were carried out using SPSS version 22.0 (IBM Corp. 2013). We determined characteristics of patients carrying carbapenemase-producing bacteria with 95% confidence interval (CI); odds ratio (OR) was calculated. Chi-square and Fisher exact tests were used for categorical variables. Independent sample t-test was used for comparing the mean ages of the two groups.

Real-time PCR was considered as a gold standard method. Sensitivity and specificity, negative predictive values (NPV), and positive predictive values (PPV) were also calculated. These values of MHT and CDT were compared using the nonparametric McNemar's test.* P-*values less than 0.05 were considered as statistical significance.

## 3. Results and Discussion

### 3.1. Results

#### 3.1.1. Characteristics of Carbapenemase-Producing E. coli and K. pneumoniae at 2 Hospitals in the Southern Vietnam

In general, of 100 strains from clinical samples (50 isolates of* E. coli* and 50 isolates of* K. pneumoniae*), 47 isolates were positive by real-time PCR. In those positive strains, 36 carried* bla*NDM, 10 carried* bla*KPC, and one carried both genes. 58.3% (21/36) strains carrying* bla*NDM and 80% (8/10) strains carrying* bla*KPC were* K. pneumoniae*. The one carried both genes was* E. coli*. CRE were mainly isolated from sputum (37%) and urine (31%). Samples were collected from 13 different sites of ICU (14%), Department of Urologic Surgery (14%), Infectious Disease Department (17%), Geriatrics Department (9%), Respiratory Department (9%), and some other departments (37%). The majority of the study participants were male (55%). The average age was 66.9 (95% CI: 63.6–70.9), the youngest patient was 23, and the oldest one was 94. The proportion of people aged 60 years and older was 69%; the odd ratio of patients in the elder group infected by* E. coli *or* K. pneumoniae* having carbapenemase-encoding genes was 3.18 (95% CI: 1.28–7.89) compared to the other group. Furthermore, in Dong Nai General Hospital, the number of the elders infected by NDM/KPC producers was significantly higher than that of the younger ones (p=0.001). The prevalence of pneumonia was 29.0%, the odd of CP-E/K infected patients with pneumonia was 2.32 (95% CI: 0.93–5.78) compared to CP-E/K infected patients without pneumonia; the odd of elders with pneumonia was 9.32 (95% CI: 2.05–42.29) compared to the younger ones.

**Figure 1:**
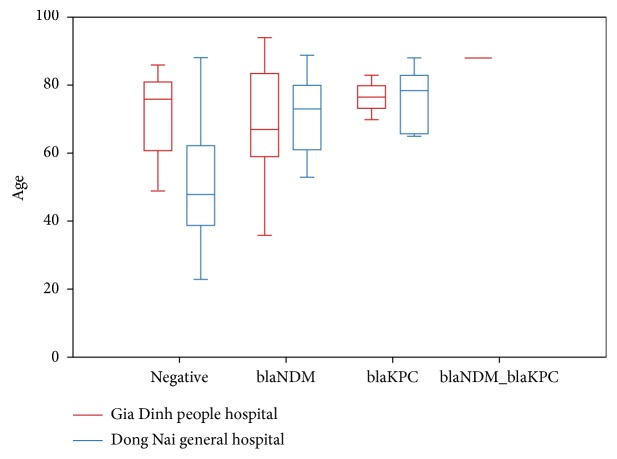
Different distribution of patients' age following genotype at 2 hospitals.

**Figure 2:**
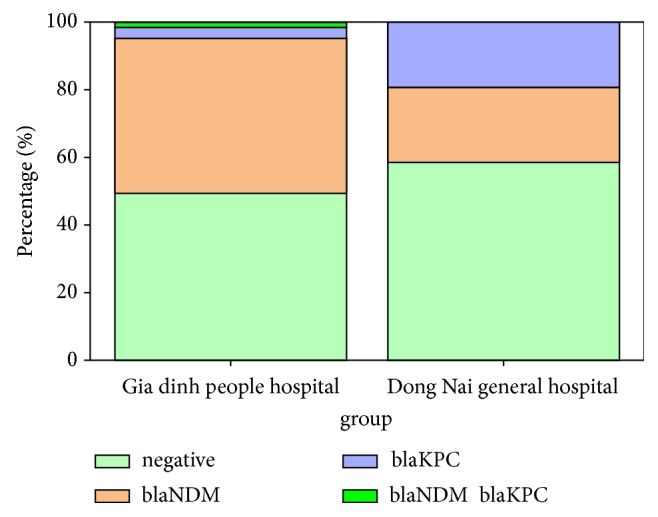
Different distribution of prevalence of CPE at 2 hospitals.

Moreover, data from our study indicated that the rate of CPE is different between the two hospitals. NDM-producing organisms were the major pathogens in Gia Dinh People's Hospital (45.8%). The prevalence of bacteria carrying* bla*NDM and* bla*KPC seems to be the same in Dong Nai General Hospital (22% for* bla*NDM versus 19.5% for* bla*KPC) (Figure 1). Other characteristics of CRE infected patients were described in Figures 1 and 2, Tables 2 and 3.

#### 3.1.2. Molecular Detection

The conformity of three methods was illustrated in Table 3.

#### 3.1.3. The Modified Hodge Test (MHT)

All isolates were evaluated by using the MHT and real-time PCR. This method showed low sensitivity and specificity compared to real-time PCR, especially in* K. pneumoniae.* 38% (23/53) of strains were positive with the MHT whereas the real-time PCR results were negative. 8/47 strains were real-time PCR positive but negative for the MHT, and 7 of those 8 strains carried* bla*NDM. The sensitivity and specificity for* E. coli* and* K. pneumoniae *were 72.2%, 68.8% and 89.1%, 38.1%, respectively. Moreover, the PPV and NPV for* E. coli* and* K. pneumoniae *were 56.5%, 81.5% and 66.7 %, 72.7%, respectively.

#### 3.1.4. Combined Disk Test

In the same way, all isolates were tested using combined disk test (using EDTA and PBA).

### 3.2. Discussion

In our study, the prevalence of NDM and KPC-producing bacteria in both hospitals is high. Furthermore, the figures of NDM and KPC-producing E/K among infected patients were different between the two hospitals, which depend on areas and ages of patients (Figure 2). Probably, the high rate of integron and gene cassette carrying* E. coli *at Gia Dinh People's Hospital (45/65 strains carrying intergron gene and 32 of these 45 intergron harboring strains carrying gene cassette) [34] led to coharboring of the two genes in* E. coli *strain. In particular, this is the first report of the presence of* E. coli* strain coharboring both genes in Vietnam.

The average age of patients and the proportion of elder group at Gia Dinh People's Hospital were higher than these figures for Dong Nai General Hospital. In Dong Nai General Hospital, people infected by non-NDM/KPC-producing bacteria were young, most of whom were the group in the range from 40 to 60 (Figure 1); these young people could be infected with ESBL/AmpC producing* E. coli/K. pneumoniae*. Another hypothesis might be that the isolates from these patients could carry other carbapenemase-encoding genes, such as* bla*OXA-48-like, but these mechanisms cause a lower level of resistance [2, 17, 18]. These features could play an important role in the difference of the odd of positive and negative outcome of the elder group between these 2 hospitals. The elders who suffered from pneumonia and infection with the two types of carbapenemase producers were more than the younger ones, especially in Dong Nai General Hospital. The reason for the difference between these two hospitals was still unclear. It could be explained that Gia Dinh People's Hospital was established for a long time; the severity of illness was quite different compared with Dong Nai General Hospital.

Regarding the methods, real-time PCR is an accurate and rapid method to detect the presence of carbapenemase-encoding genes. However, the cost associated with the method is one of the major disadvantages that limits its use in common laboratories in Vietnam. Among various phenotypic methods, MHT recommended in CLSI 2017 is a simple method [33]. In this study, SPC of MHT is low (23/53* K. pneumoniae strains *were positive, but negative for the real-time PCR). The possible reason for this is that other types of carbapenemase may present in these bacteria. However, it could be explained that AmpC, ESBLs, or strict requirements of MHT process at each step may cause false positive results [35]. Besides, this method shows low sensitivity to NDM-producing Enterobacteriaceae (7/8 strains were found to have a false-negative result) [36, 37]. In our study, the MHT results for* K. pneumoniae *are consistent with findings in recent studies conducted by Dariush Shokri et al. [38] and Rangneka R aSeeM et al. [39]. This may be the reason why the CLSI 2018 standard excluded MHT in the guideline [40].

In contrast, CDT has been shown to be highly effective. The results of NDM detection by EDTA disk test for* K. pneumoniae *(Table 4) were in accordance with findings obtained by Paradimitriou–Oliveris et al. [30]. However, it was in contrast to findings reported by Dariush Shokri et al. [38]. This could be explained by the fact that Shokri carried out his study including not only* K. pneumoniae*, but also* A. baumanii* and* P. aeruginosa*. The high rate of* bla*VIM in* P. aeruginosa *may be the reason for false-negative results. A study conducted by Ting-ting Qu et al. in 2009 showed that CDT also detected other types of carbapenemase including* Verona* integron-borne metallo-*β*-lactamase (VIM), which was the most widespread metallo-*β*-lactamase in* P. aeruginosa* [41, 42]. Our results for KPC detection using PBA disk test* K. pneumoniae *(Table 4) were in accordance with findings obtained by Athanasios Tsakris et al. (using PBA-400*μ*g) [31] and Julio Zúñiga et al. (using 300*μ*g APBA) [43]. The efficiency of PBA disk test in* E. coli *was low, particularly in sensitivity and PPV because of the sample size of KPC-producing* E. coli*, only 3 strains with one of them being a false negative. The results in Tables 5 and 6 illustrated the significantly higher specificity of combined disk test compared with MHT.

## 4. Conclusions

The prevalence of CP-E/K in Vietnam is high. The lack of suitable methods to detect and differentiate these bacterial infections has been the main obstacle for clinicians to choosing an appropriate treatment. During this study, we recognized that CDT is reasonable and simple to detect the presence of these important enzymes. These features make it highly applicable to all clinical laboratories in Vietnam.

## Figures and Tables

**Table 1:** Primers and probes used in this study.

Oligonucleotide	Nucleotide sequence, 5′–3′	Origin	Volume of reaction (nM)	Reference

*Primers and probes for real-time PCR*				

NDM-F Primer	GAC CGC CCA GAT CCT CAA	IDT	300	
NDM-R Primer	CGC GAC CGG CAG GTT	IDT	300	
NDM-probe	HEX-TG GAT CAA GCA GGA GAT-ZEN/IBFQ	IDT	200	
KPC-F Primer	GGC CGC CGT GCA ATA C	IDT	500	
KPC-R Primer	GCC GCC CAA CTC CTT CA	IDT	500	[32]
KPC- probe	6FAM-TG ATA ACG CCG CCG CCA ATT TGT-ZEN/IBFQ	IDT	200	
16S rRNA-F Primer	TGG AGC ATG TGG TTT AAT TCG A	IDT	200	
16S rRNA-R Primer	TGC GGG ACT TAA CCC AAC A	IDT	200	
16S rRNA-probe	Cy5-CA CGA GCT GAC GAC AR*∗*C CAT GCA-IBRQ	IDT	100	

*Primers for DNA sequencing*				

NDM-F-seq	AGT CGC TTC CAA CGG TTT	IDT	300	This study
NDM-R-seq	CAT TGG CAT AAG TCG CAA TCC	IDT	300	
KPC-F-seq	GGT CAC CCA TCT CGG AAA	IDT	500	
KPC-R-seq	GGG ATG GCG GAG TTC AG	IDT	500	

**Table 2:** Distribution of diseases.

Hospital		Disease								Total
		Pneumonia	UTI	Wound Infection	Abnormal Infection	Bacteremia	Fever	Surgical Wound Infection	Other	
GD (N=59)	E	2	14	5	0	5	2	0	1	29
	K	13	9	2	1	2	2	1	0	30

DN (N=41)	E	3	10	3	0	0	1	2	2	21
	K	11	2	3	0	0	0	2	2	20

Total		29	35	13	1	7	5	5	5	100

*∗*E: *E. coli*, K: *K. pneumoniae*, GD: Gia Dinh People's Hospital, DN: Dong Nai General Hospital, UTI: Urinary Tract Infection.

**Table 3:** Particular characteristics of patients at 2 hospitals in this study.

Characters	Gia Dinh People's Hospital (n=59)	Dong Nai General Hospital (n=41)	*P*-value
Average age (95% CI)	71.5 (67.4 – 75.2)	60.4 (52.2 – 66)	0.003
Range	36 – 94	23 – 89	
Elder (%)	76.3	58.5	0.079
Positive rate (%)	50.8	41.5	0.545
Pneumonia rate (%)	25.4	34.1	
Odd of Outcome (Pos/Neg) between Pneumonia and the other	0.79	7.14	
Odd of Pneumonia (Elder/Younger)	5.88	18.91	
Odd of Outcome (Positive/Negative) in Elder group/ the other.	1.045	15	
Therapy departments (%)	ICU (20.3), Geriatrics (15.3), Department of Urologic surgery (10.2), Respiratory Department (8.5), and other departments (45.7)	Infectious Disease Department (41.4) Department of surgery (19.5), Respiratory Department (9.8), ICU (4.9), and other departments (24.4)	
*K. pneumoniae* (%)	50.8	48.8	

**Table 4:** The agreement of bacteria isolation, real-time PCR, and DNA sequencing results.

Strains ID	Isolation	Real-time PCR	DNA sequencing
GD013	*K. pneumonia*	*bla*NDM, 16s rRNA	*bla*NDM, *K. pneumoniae*
GD002	*E. coli*	*bla*NDM	*bla*NDM
GD018	*E. coli*	*bla*NDM, *bla*KPC, 16S rRNA	*bla*NDM, *bla*KPC, *E. coli*
GD011	*K. pneumoniae*	*bla*KPC	*bla*KPC
GD016	*K. pneumoniae*	*bla*KPC	*bla*KPC
DN011	*K. pneumoniae*	*bla*KPC	*bla*KPC

**Table 5:** Combined disk test results in *E. coli *and *K. pneumonia.*

Combined disk test			Real-time PCR		TPR (%) 95%	SPC (%)	PPV (%)	NPV (%)	Accuracy (%)
	Species		Pos	Ne					
NDM detection	*E. coli* (n=50)	Pos	15	1	93.8	97.1	93.8	97.1	96.0
		Neg	1	33					
	*K. pneumoniae* (n=50)	Pos	19	0	90.5	100	100	93.6	96
		Neg	2	29					

KPC detection	*E. coli* (n=50)	Pos	2	2	66.7	95.7	50	97.8	94.0
		Neg	1	45					
	*K. pneumoniae* (n=50)	Pos	8	3	100	92.9	72.7	100	94
		Neg	0	39					

*∗*Pos: positive, Neg: negative.

**Table 6:** Comparison between the MHT and combined disk test.

Bacteria	Index	NDM detection			KPC detection		
		*MHT*	*EDTA*	*McNemar∗*	*MHT*	*Boronic*	*McNemar∗*
*E. coli *(n=50)	TPR (%)	72.2	93.8	p=0.25	72.2	66.7	p=1.00
	SPC (%)	68.8	97.1	p=0.006	68.8	95.7	p<0.001

*K. pneumoniae *(n=50)	TPR (%)	89.7	90.5	p=1.00	89.7	100	NC
	SPC (%)	38.1	100	p<0.001	38.1	92.9	p<0.001

*∗* means nonparametric McNemar test.

## Data Availability

The data used to support the findings of this study are available from the corresponding author upon request.

## Additional Points

*Limitation*. In our study, other genes such an OXA-48-like, ESBL, and AmpC *β*-lactamase were not included, so this could affect our study results. The only 10 KPC-producing organisms in this study may have effects on the results of PBA disk test. A lack of genotypic method to identify* E.coli* and* K. pneumoniae *was also important.

## Supplementary Materials

Supplementary Materials List of related information of patients and testing results were detailed in Annex 1. Data analysis results, testing images, reference, and full-text of gray literature, were described in Annexes 2, 3, 4, 5, respectively.
